# Anni 2.0: a multipurpose text-mining tool for the life sciences

**DOI:** 10.1186/gb-2008-9-6-r96

**Published:** 2008-06-12

**Authors:** Rob Jelier, Martijn J Schuemie, Antoine Veldhoven, Lambert CJ Dorssers, Guido Jenster, Jan A Kors

**Affiliations:** 1 Department of Medical Informatics, Erasmus MC University Medical Center, Dr. Molewaterplein, Rotterdam, 3015 GE, The Netherlands; 2 Department of Pathology, Erasmus MC University Medical Center, Dr. Molewaterplein, Rotterdam, 3015 GE, The Netherlands; 3 Department of Urology, Erasmus MC University Medical Center, Dr. Molewaterplein, Rotterdam, 3015 GE, The Netherlands

## Abstract

Anni 2.0 is an online tool () to aid the biomedical researcher with a broad range of information needs. Anni provides an ontology-based interface to MEDLINE and retrieves documents and associations for several classes of biomedical concepts, including genes, drugs and diseases, with established text-mining technology. In this article we illustrate Anni's usability by applying the tool to two use cases: interpretation of a set of differentially expressed genes, and literature-based knowledge discovery.

## Abstract 2

Anni 2.0 provides an ontology-based interface to MEDLINE.

## Rationale

The amount of biomedical literature is vast and growing rapidly. It has become impossible for researchers to read all publications in their field of interest, which forces them to make a stringent selection of relevant articles to read. To keep abreast of the available knowledge, a wide range of initiatives has been deployed to mine the literature, from manual encoding of gene relations by the Gene Ontology Consortium [1], to automatic extraction of specific information such as transcript diversity [2], to the use of literature data for the prediction of disease genes [3,4] (see [5,6] for recent reviews). One of the emerging approaches is text-mining, which infers associations between biomedical entities by combining information from multiple papers. Text-mining approaches typically rely on occurrence and co-occurrence statistics of terms and have been successfully applied to a number of problems. The classic application is for literature-based knowledge discovery, which attempts to link disjunct sets of literature in order to derive promising new hypotheses [7-11]. Swanson (see, for example, [12]) was a pioneer in this field and was able to publish several new hypotheses derived with the help of literature mining. His well known first example was the hypothesis that Raynaud's disease could be treated with fish oil [13], which was later corroborated experimentally [14]. Another field to which text-mining has been successfully applied is the analysis of DNA microarray data [15-17]. With microarray experiments, hundreds of genes can be identified that are relevant to the studied phenomenon. The interpretation of such gene lists is challenging as, for a single gene, there can be hundreds or even thousands of articles pertaining to the gene's function. Text-mining can alleviate this complication by revealing the associations between the genes that are apparent from literature. This was the focus of the earlier version of Anni [18].

**Figure 1:**
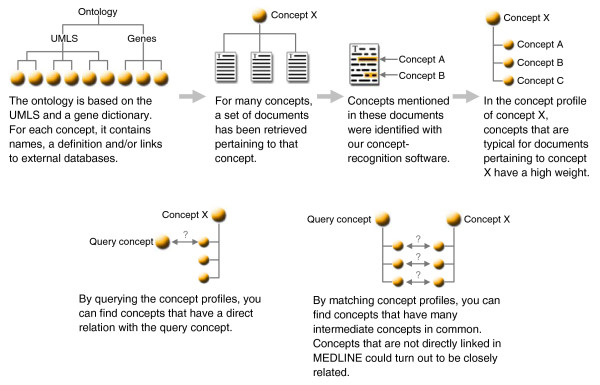
The technology behind Anni at a glance. Yellow balls indicate ontology concepts.

Here we present Anni 2.0, a tool that provides an ontology-based interface to the literature. The tool is aimed at a broad audience of biomedical researchers and facilitates traversing the huge corpus of biomedical literature efficiently to answer a broad range of information needs, including those for the interpretation of high-throughput datasets. Anni's functionality is based on the use of an ontology, which defines concepts, such as genes, biological processes and diseases, and their relations. Concepts come with a definition, a semantic type, and a list of synonymous terms and can be linked to online databases. We identify references to concepts in texts with our concept recognition software Peregrine [19]. The idea behind Anni is to relate or associate concepts to each other based on their associated sets of texts. Texts can be linked to a concept through automatic concept recognition, but also by using manually curated annotation databases. The texts associated with a concept are characterized by a so-called concept profile [18] (see Figure 1 for an introduction into the technology behind Anni). A concept profile consists of a list of related concepts and each concept in the profile has a weight to signify its importance. Concept profiles have been successfully used to infer functional associations between genes [18,20] and between genes and Gene Ontology (GO) codes [21] to infer novel genes associated with the nucleolus [22], and to identify new uses for drugs and other substances in the treatment of diseases [8].

Anni 2.0 provides a generic framework to explore concept profiles and facilitates a broad range of tasks, including literature based knowledge discovery. The tool provides concepts and concept profiles covering the full scope of the Unified Medical Language System (UMLS) [23], a biomedical ontology. The user is given extensive control to query for direct associations (based on co-occurrences), to match concept profiles, and to explore the results in several ways, for instance with hierarchical clustering. Several types of ontological relations can be used in Anni. Semantic type information, which indicates whether a concept is about, for example, a gene or a drug, can be used to group concepts. This allows, for instance, a query as to whether a gene of interest has an association with any of the available diseases. Hierarchical 'parent/child' relations are also available and can be visualized. They can be used to explore the relations in a group of concepts or to expand a query by identifying relevant related concepts in the hierarchy. An important feature of Anni is transparency: all associations can be traced back to the supporting documents. In this way, Anni can also be used to retrieve documents about concepts of interest, thereby exploiting the mapping of synonyms and the resolution of ambiguous terms by our concept recognition software.

Previously, we illustrated the utility of concept profiles to retrieve functional and relevant associations between various types of concepts [18,21,22]. Here, we evaluate our tool through two use cases. First we use Anni to analyze a DNA microarray dataset. Second, we attempt to reproduce and expand a published literature-based knowledge discovery.

## Implementation

### Information sources

Anni is a Java client-server application and communicates with our server through remote method invocation. It uses three information sources.

One source is an ontology composed of the 2006AC version of the UMLS ontology [23] and a gene thesaurus derived from multiple databases [24]. Following Aronson [25], the UMLS thesaurus was adapted for efficient natural language processing, avoiding overly ambiguous or duplicate terms, and terms that are very unlikely to be found in natural text. The gene thesaurus contains genes from three species: human, mouse and rat. Homologs from these three species were mapped through NCBI's Homologene database [26]. In addition, genes with identical nomenclature were mapped to each other.

A second source is a database with indexed textual references to ontology concepts in MEDLINE abstracts (from 1980 on). For concept recognition, we make use of our Peregrine software [19]. Apart from mapping synonomous terms to one concept as identified by the ontology, Peregrine attempts to disambiguate words or phrases that refer to multiple terms based on contextual information. Participation in the Biocreative 2 competition shows Peregrine can recognize genes and proteins in text with a precision of 75% and recall of 76%, making it comparable to the current state-of-the-art. Abstracts were indexed together with the medical subject headings (MESH) concepts. MESH is a controlled vocabulary and concepts are manually assigned to abstracts to facilitate document retrieval. The registry number field (RN field) contains information on chemicals to which the abstract refers and was also incorporated in the analysis. The recall of the recognition of references to genes in texts was increased by taking common spelling variations into account [27].

A third source is a database with concept profiles based on the MEDLINE indexation. The basis of a concept profile is a set of abstracts associated to a concept. For GO terms we used the papers associated with the term by the GO annotation consortium [1]. For genes the set of abstracts in which the gene occurs was taken, but from a subset of MEDLINE containing documents on mammalian genes, selected by the PubMed query "(gene OR protein) AND mammals". For the other concepts we relied on the complete MEDLINE indexation. The weights in the concept profiles were derived by means of the symmetric uncertainty coefficient [28] (see [21] for a study on weighting schemes for concept profiles). For efficiency, we excluded from the concept profiles concepts with an association score lower than 10^-8 ^and concepts that occurred only once in the MEDLINE indexation.

### Design paradigms

Anni is organized through concept sets, which are displayed in a tree view. Upon startup a range of predefined concept sets are loaded: the three branches of the GO [29], the set of genes, and the semantic types as defined by the UMLS, for example, "Disease or Syndrome" or "Biologically Active Substance". Users can manipulate concept sets through basic set operations such as intersection, union and substraction, or they can create a new concept set and add concepts through an input panel. With the input panel the user can provide concept names or identifiers from several databases (Entrez Gene, Swiss-Prot and Gene Ontology identifiers, among others) through typing, pasting or loading a text file, and map them to concepts. To explore hierarchical relations between the concepts in a concept set, the concepts can be shown in a relational tree view.

Wherever in the application concepts are shown, they can be selected and, through a dropdown menu, several options are available: show concept definition and semantic types; transfer concepts to a new concept set; show concept profile (if available).

In Anni, many concepts have a concept profile. Concept profiles can be both queried and matched. A query on concept profiles will retrieve concept association scores based on the concepts' co-occurrences, for example, a query with the concept "prostate cancer" on the set of all genes will retrieve the genes mentioned together with this concept in abstracts, sorted by strength of association as measured by the uncertainty coefficient. Queries are performed with a query concept profile and query concepts can be individually weighted by the user. The table with the query results allows the user to sort on concept profiles that contained all the query concepts. In addition, the co-occurrence rate between concepts as observed in the MEDLINE database can be shown in the query result table. The query result table can be explored through two-dimensional hierarchical clustering and a heatmap.

**Figure 2:**
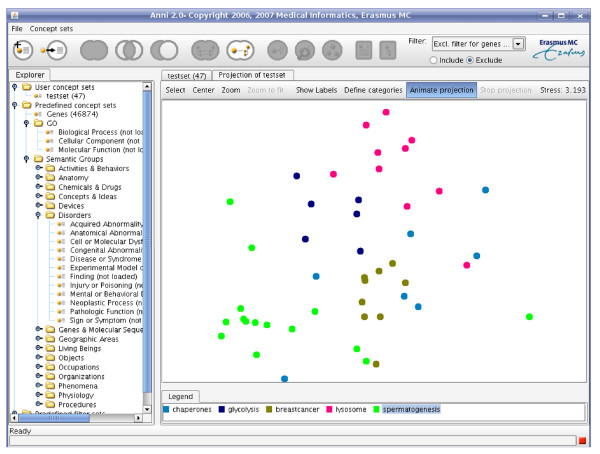
Screenshot of Anni. An MDS projection is shown of a test set of 47 genes, organized in 5 groups through a shared commonality (see legend and [17,18]). In the Explorer tab to the left, concept sets are organized in a tree. The toolbar on top provides concept set options and shows the current filter for matching concept profiles. The shown MDS view on a concept set can be used to get an overview of associations between the concepts, as used, for instance, in [22]. Groups of nodes can be selected and the similarities between their concept profiles analyzed in the annotation view. Nodes are colored based on user-defined features.

Concept profiles can be matched to identify similarities between concept profiles, for instance, to identify genes associated with similar biological processes. As a matching score we use a scaled inner product score between concept profiles. The user can use a filter to control which concepts are used for matching. Concept sets can be used as an inclusive filter - only the concepts in the concept set are used for matching - or as an exclusive filter - all concepts are used for matching except the concepts in the filter concept set. The associations between concept profiles within a concept set can be explored through hierarchical clustering or a multi-dimensional scaling (MDS) projection (Figure 2). Additionally, two concept sets can be matched, which will result in a matrix of association values. Similar to the query result table, the direct co-occurrence frequency can be shown. Concepts with a high association score but no MEDLINE co-occurrences could indicate a new discovery: an association between concepts implicit in the literature but not yet explicitly described. The matrix can also be explored through two-dimensional hierarchical clustering and a heatmap.

To provide transparency, Anni is equipped with an annotation view to evaluate the similarity within a group of concept profiles. The view provides a coherence measure, the average of the inner product scores of all possible pairs within the group. To aid the interpretation of the inner product scores, the probability is given that the same score or higher would be found in a randomly formed group of the same size. In addition, the percentage of the contributions of individual concepts to the coherence score are shown as well as the weights of these concepts in the individual concept profiles. Finally, every association in a concept profile can be traced to the supporting documents.

## Results

### Use case 1: analysis of a DNA microarray dataset

For this use case we applied Anni 2.0 to analyze a set of genes differentially expressed between localized and metastasized prostate cancer to unravel genes and pathways responsible for the progression of prostate cancer to metastatic disease. The dataset was generated based on three published studies [30-32]. Data from these studies were processed as in the original papers. For inclusion in our set, genes had to be in the top differentially expressed genes in at least two of the three studies. The set contained 69 genes expressed higher in metastasized cancer compared to local prostate cancer and 130 genes with lower expression (Additional data file 2). As a first step we investigated if there were genes known to be associated with prostate cancer. We performed a query for the concept "malignant neoplasm of the prostate". Sixty-eight genes had a direct association through co-occurrence, which is a highly significant over-representation (*p *= 2.04 × 10^-8^) given the number of genes associated with this concept in the predefined concept set "Genes".

**Figure 3:**
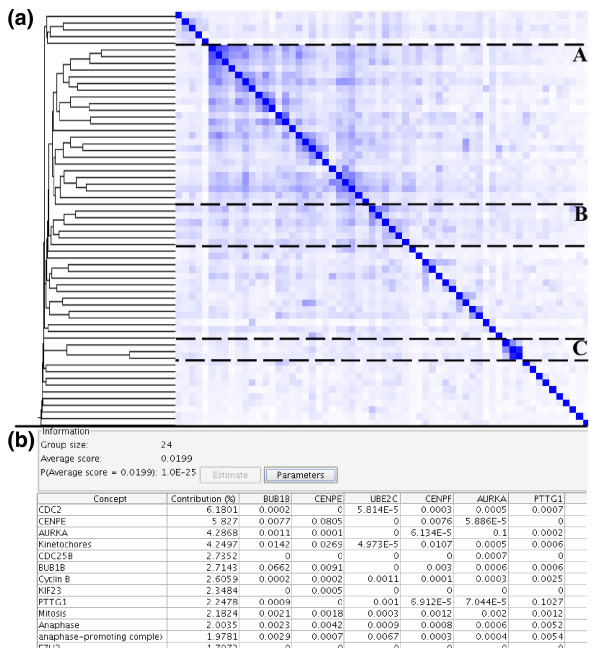
Clustering and annotation of differentially expressed genes. **(a) **The clustering of genes up-regulated in prostate metastases. The clustering is based on the similarity of the concept profiles of the genes. **(b) **A fragment of the annotation for cluster A. The annotation view displays for a cluster a group cohesion score with a *p*-value, and a list of concepts with their percentage contribution to the score. In addition, the weights of the concepts in the concept profiles are shown.

To identify shared associated concepts between the genes in general, we clustered the up- and down-regulated genes separately. During the matching a broad semantic filter was employed to select for biomedical concepts relevant for gene function [18] (the filter is included as a predefined concept set). Figure 3 shows the clustering for genes more highly expressed in metastatic prostate cancer and Table 1 shows all identified clusters (for the full annotation see Additional data file 2). First, we consider the analysis of genes down-regulated in metastases. Two of the clusters are characterized by concepts apparently pertaining to the prostate stroma, such as "smooth muscle myosins" and "extracellular matrix proteins". This is expected as organ confined tumors contain stroma, whereas metastases, mainly from lymph nodes, are free of prostate stromal cells. Other gene clusters with lower expression in metastases pertain to the level of differentiation of the cancer cells and hence the grade of the cancer. Lower grade prostate tumors contain more differentiated epithelial cells that are involved in the secretion of prostatic fluid, which is reflected by clusters characterized by concepts such as "membrane transport proteins" and "exocytosis" [18].

**Table 1:** A selection of identified relevant clusters in the set of differentially expressed genes between metastatic and localized prostate cancer

Cluster	Number of genes	Descriptive concepts
Up-regulated		
A	24	Kinetochores; mitosis; anaphase-promoting complex
B	5	Nuclear proteins; tumor markers, biological
C	3	Unfolded protein response
		
Down-regulated		
A	7	Complement system proteins
B	7	Calponin; smooth muscle myosins
C	4	Myosin phosphatase; smooth muscle (tissue)
D	7	Extracellular matrix proteins
E	5	Transcription factor; proto-oncogene proteins c-fos
F	4	Cyclin-dependent kinases
G	3	Melanosomes; membrane protein traffic; exocytosis
H	5	Membrane transport proteins; symporter

The most descriptive concepts are shown as given by the Anni annotation view. The two left-most columns depict how many genes in the cluster were either up- or down-regulated in metastasized prostate cancer.

The clustering of genes more highly expressed in metastatic prostate cancer is dominated by the large cluster associated with kinetochores, anaphase-promoting complex and mitosis (Figure 3). In this cluster, subclusters associated with "kinetochores", "mitotic checkpoint" and "anaphase promoting complex" indicate the cluster is not just a signature of proliferation, but shows associations with a specific phase in mitosis: the spindle checkpoint. Indeed, the concept "spindle checkpoint activity" was the 13th concept (not counting genes) in the annotation for this cluster. The spindle checkpoint prevents a dividing cell from advancing from metaphase into anaphase before all kinetochores are correctly attached to the mitotic spindles. A kinetochore is the protein structure assembled on the centromere that links the chromosome to the microtubules of the mitotic spindle. The anaphase promoting complex (APC) ubiquitin ligase plays an important role in controlling the progression to anaphase by triggering the appropriately timed, ubiquitin-dependent proteolysis of mitotic regulatory proteins. A perturbation involving the APC is apparent, as a query on "anaphase promoting complex" reveals that 11 of the up-regulated genes have a strong association (>10^-5^), which is a highly significant overrepresentation (*p *< 5 × 10^-11^). Using the links in the application to the underlying literature and the Entrez Gene database, we can easily confirm the associations. For instance, for the genes shown in Figure 3b, CENPE is a kinetochore protein and CENPF is essential for kinetochore attachment [33], BUB1B is a mitotic checkpoint protein interacting with the APC [34], PTTG1 and AURKA are substrates of the APC [35,36] and UBE2C is one of the two ubiquitin-conjugating enzymes used by the APC [37,38]. All retrieved associations discussed above were supported by a set of supporting documents that was partially composed of documents predating the earliest microarray experiment publication, that is, they do not only reflect recent findings.

Deregulation of APC *in vitro *can result in defects in chromosome segregation, chromosomal instability, aneuploidy and increased sensitivity for tumorigenesis (for a review, see [39]). Also, changed levels of APC regulators and substrates have been found to be correlated with cancer malignancy and, for some cancers, with tumor aggressiveness [40]. A causal relation between deregulation of APC and malignancy or tumor aggressiveness has been suggested to exist through a higher mutation rate. Nevertheless, causality is not established *in vivo*, and observed APC deregulation could also be a consequence of tumorigenesis and genomic instability. Interestingly, Lehman *et al*. [40] did not find an APC mitotic cluster in prostate cancer and attributed this observation to the low aggressiveness of prostate cancers. As they studied organ confined prostate cancer, this is in line with our observation here. It appears, therefore, that also in prostate cancer, APC deregulation is correlated with tumor aggressiveness. Deregulation of the APC could have clinical consequences as some anti-neoplastic agents, such as nocodazole and taxol, work by activation of the spindle checkpoint [40]. Deregulation of the APC could, therefore, reduce the effectiveness of these drugs. For instance, overexpression of UBE2C can cause the nocodazole induced mitotic blockade to be bypassed [41].

Concluding, with Anni we were able to functionally annotate a DNA microarray dataset. Genes published as associated with prostate cancer were easily retrieved. We identified clusters with genes with lower expression levels in metastases likely associated with stroma and differentiation features of cancer cells. Among the genes more highly expressed in metastases, we identified a cluster associated with the spindle checkpoint and the APC. This is a previously unknown feature of metastasized prostate cancer and may be an indicator of the aggressiveness of the cancer.

### Use case 2: literature-based knowledge discovery

Here, we illustrate Anni's knowledge discovery potential by reproducing a published literature-derived hypothesis. When looking for new therapeutic uses of the drug thalidomide, Weeber *et al*. [7] suggested, amongst others, that chronic hepatitis C could be treated with thalidomide. We selected this hypothesis as experimental evidence has recently emerged that appears to substantiate the claim [42,43]. Weeber *et al*. took the following approach: first, from the MEDLINE database concepts of the UMLS semantic type "immunological factors" were automatically retrieved that occurred together in a sentence with thalidomide. At position 7 in their list they found the concept "interleukin-12". Through the association of this concept with thalidomide, they identified an interesting biological process modulated by thalidomide. Second, they queried concepts of the semantic type "Disease or Syndrome" for association with the selected process of interest. Third, from the query results, diseases known to be associated with thalidomide were automatically removed and, after some additional manual curation, a shortlist was analyzed by an expert to identify diseases that could benefit from thalidomide treatment.

For reproducing this experiment we used the set of MEDLINE records published up to the time point given by Weeber *et al*. (July 2000), and generated concept profiles based on this set of records. In three simple steps, and closely following the considerations mentioned in the original article, we could reproduce Weeber *et al*.'s query. In the first step, based on the predefined concept sets available in Anni, we can readily select concepts belonging to a semantic type of choice. To reproduce Weeber *et al*.'s first filtering, we selected the predefined concept sets "Genes" and "Immunological factors", merged them and set the resulting set as an inclusive filter (we include "Genes" because genes in the UMLS thesaurus were removed in favor of our custom made gene thesaurus). With this filter, "interleukin-12" has a high rank in the concept profile of thalidomide - coincidentally, also seventh - which reproduces the first step of their approach.

As the next step, we queried the 8,152 concepts of the predefined concept set "Disease or Syndrome" for which a concept profile is available. Weeber *et al*. [7] describe the biological process they queried as follows: "Thalidomide has strong inhibitory effects on mononuclear cell production of IL-12 and a stimulatory effect on IL-10 production." Through these effects, thalidomide influences the balance of T-helper 1 versus T-helper 2 cells. Based on this description, we generated the following query: "IL-12", "IL-10", "Th1 cells", "Th2 cells" and "peripheral mononuclear cells". All concepts in the query were given equal weight, and all concepts were required to occur in the disease concept profile.

As we are only interested in diseases not previously associated with thalidomide, in the third step all diseases mentioned with thalidomide in a MEDLINE record (up to July 2000), were removed automatically from the resulting ranking (the query view can show MEDLINE co-occurrence rates). After this, some simple and straightforward additional manual cleanup was performed on the query result to create a shortlist for the expert: diseases closely related to previously filtered diseases that had a known association with thalidomide were removed - for example, "severe combined immunodeficiency" was removed since thalidomide has been used to treat wasting in AIDS; impractically broad disease concepts were removed, such as "parasitic infection"; closely related diseases were mapped to a single disease to reduce redundancy - for example, "cutaneous leishmaniasis", "leishmaniasis" and "visceral leishmaniasis" were mapped to "leishmaniasis"; and animal diseases were removed, for example, "toxoplasmosis, animal". The filtering process is facilitated by viewing the hierarchical relations between the concepts in Anni.

The top ten of our results are shown in Table 2; chronic hepatitis C appears sixth. Interestingly, of the higher scoring diseases, we found that PubMed now contains preliminary studies on the use of thalidomide for the treatment of leishmaniasis [44] and listeriosis [45]. On closer inspection, an association between leishmaniasis could actually have been found before 2000, because the parasite underlying the disease, *Leishmania*, had been mentioned in connection with thalidomide [46].

**Table 2:** Final ranking of diseases for use case 2

Rank	Disease name	Score
1	Leishmaniasis	0.002417946
2	*Schistosoma mansonii *infection	5.68E-04
3	Extrinsic asthma	5.44E-04
4	Listeriosis	4.88E-04
5	HTLV-I infections	3.44E-04
6	Hepatitis C, chronic	3.43E-04
7	Tropical spastic paraparesis	3.17E-04
8	Epstein-Barr virus infections	2.73E-04
9	Hepatitis B, chronic	2.38E-04
10	Filarial elephantiases	2.38E-04

Final ranking and scores for the query for "IL-12", "IL-10", "Th1 cells", "Th2 cells" and "peripheral mononuclear cells" on the concept set "Diseases or Syndromes".

## Discussion

With Anni we make available to the public a text mining methodology that we have successfully applied to several tasks: retrieving associations between genes, the functional annotation of genes, the functional annotation of the nucleolar proteome and the prediction of novel nucleolar proteins [18,21,22]. In this report Anni was applied to two very different use cases with good results: a new hypothesis on the progression of localized prostate cancer to metastatic disease and reproduction and extension of a previously published literature-based discovery. The tool has several innovative and useful features as described below.

Anni uses a concept-based approach. Definitions for the concepts are available in the application, as well as links to external databases and ontological information such as semantic type and 'parent/child' relations. In addition, when references to concepts are identified in texts, synonymous terms are mapped to the same concept. For this process, we pursued a high level of precision through a carefully curated ontology and by applying automatic homonym disambiguation (see [19] for a system description and performance evaluation). This is especially relevant for genes, as gene terminology is rich in synonymous and ambiguous terms [47,48] and is also an important feature of information retrieval tools like iHop [49].

Anni can compare concepts based on similarities in the documents associated with these concepts; therefore, implicit relations between concepts can be found. In addition, the user has complete control over which concepts are taken into account during the comparison. Combined, these features are very useful for knowledge discovery [8]. The approach also allows concepts to be included that are very hard to find in documents, such as GO codes, which are usually described with long, systematic terms.

Anni is a highly interactive application and offers a range of options to interactively explore the implicit and explicit associations between concepts. Query and match results can be viewed in a textual representation or in a graphical form through hierachically clustered heatmap or MDS projection visualizations. In addition, the tool provides a high level of transparency, which further improves its use.

Anni is a multi-purpose text-mining tool and the modular set-up and broad range of biomedical concepts allow many more tasks than the ones presented. The broad applicability of Anni 2.0 contrasts strongly with the majority of the previously published text-mining tools as well as with the earlier version of Anni. Text-mining tools tend to focus on one application, such as knowledge discovery [11,50] or the analysis of DNA microarray data [16,18,20]. Arrowsmith [11], for example, can compare two document sets to each other at a time, which is well suited for knowledge discovery, but impractical when looking for associations between a group of genes. TXTgate [20] is well suited to explore indirect associations between genes, but is not suitable for knowledge discovery purposes, as it cannot compare genes to a set of diseases or drugs. To further illustrate this point, the table in Additional data file 1 provides a comparison of Anni 2.0 to 13 previously published tools.

The Anni system has some limitations. First of all, the system works with co-occurrence based associations. These associations may not always reflect functional relations or facts. In addition, Anni relies on an ontology and automatic concept recognition in texts and neither are error free. For these reasons Anni was built to be transparent and all results can be traced back to the underlying documents. Another limitation is that only genes from mouse, rat and human are covered; support for other species is in development.

In conclusion, Anni provides an innovative ontology-based interface to the literature, and builds on advanced and well evaluated text-mining technology. Anni is a highly versatile tool, applicable to a broad range of tasks. It is freely available online [51].

## Abbreviations

APC, anaphase promoting complex; GO, Gene Ontology; MDS, multi-dimensional scaling; MESH, medical subject headings; UMLS, Unified Medical Language System.

## Authors' contributions

RJ conceived of the methodology and the evaluation, generated the data, wrote the paper and contributed to programming the application. MS conceived of the user interface and contributed to the programming and the manuscript. AV contributed to the software, especially the internet communication. GJ contributed the first use case, and together with LD provided user feedback and contributed to the manuscript. JK supervised the work and revised the manuscript.

## Additional data files

The following additional data are available. Additional data file 1 is a table presenting an overview of published text-mining tools, including Anni 2.0, and their functionality. Additional data file 2 is an Excel format datasheet listing the differentially expressed genes between localized and metastasized prostate cancer as used for use case 1.

## Supplementary Material

Additional data file 1 Overview of published text-mining tools, including Anni 2.0, and their functionality. Click here for file

Additional data file 2 Differentially expressed genes between localized and metastasized prostate cancer as used for use case 1. Click here for file
