# Supplementary material for: Anni 2.0: a multipurpose text-mining tool for the life sciences
Source: Genome Biol. 2008 Jun 12;9(6):R96. doi: 10.1186/gb-2008-9-6-r96 (PMC2481428; doi:10.1186/gb-2008-9-6-r96)
Supplement: Additional data file 1 — Overview of published text-mining tools, including Anni 2.0, and their functionality. [file gb-2008-9-6-r96-S1.pdf]

Additional data file 1

This data file provides an overview of the published text-mining tools, including Anni 2.0, and their functionality. The tools are grouped by their main functionality. The columns headed by C and O indicate whether the tool is concept-based, and whether it is operational, respectively. The numbers refer to the following tasks:

1. Summarize the literature about a concept.
2. Retrieve documents for a concept (synonyms and disambiguation).
3. Find direct associations for a query concept.
4. Find direct and indirect associations for a query concept.
5. Open knowledge discovery.
6. Closed knowledge discovery.
7. Find clusters in a set of concepts based on their associations.

The final two columns, use case 1 and 2, indicate whether we could reproduce our use cases with the applications, within a reasonable time span.

The following notes are referred to in the table:

[a] Input is a keyword PubMed query, output shows concepts identified in retrieved documents.

[b] Utilizes the manual indexation of abstracts in MEDLINE with the controlled vocabulary MESH.

[c] At the time of writing several attempts were made to use the tool, and at least one (unanswered) email was sent to the authors with a request for assistance.

[d] The documents sets are defined by a PubMed query.

[e] Requires a manual PubMed query.

[f] Categorizes sentences by relational keyword distribution.

[g] Allows the user to make a network for overview.

[h] Only for genes.

[i] Retrieves sentences.

[j] For genes and gene-drug associations.

[k] Works with profiles that characterize document sets that are user-defined through PubMed queries.

[l] The tool only works under Windows™in combination with Internet Explorer™.

[m] The application does not provide a clustering nor prioritization of relevant associations and appeared to not have been updated recently. Nevertheless, for several of the genes higher expressed in metastatic prostatic neoplasms, associations were retrieved that could be traced to APC disregulation.

[n] The required queries are possible. A formal comparison would require a cutoff in time on the literature that is being used, and this is not possible through the online tool.

Tool name	PubMed ID or citation	URL	C	O	Query options	1	2	3	4	5	6	7	Use case 1	Use case 2
Literature summarization														
EbiMed	17237098	www.ebi.ac.uk/Rebholz-srv/ebimed/	output[a]	yes	keyword-based PubMed query	yes[e]	no	yes[e]	no	no	no	no	no	no
Alibaba	16870931	alibaba.informatik.hu-berlin.de/	output[a]	yes	keyword-based PubMed query	yes[e]	no	no	no	no	no	no	no	no
Information Retrieval														
Medminer	10631500	discover.nci.nih.gov/textmining/	yes	yes	keyword query	yes[f]	yes	yes[j]	no	no	no	no	no	no
PubMed		www.pubmed.org	partly[b]	yes	keyword query	no	partly[b]	no	no	no	no	no	no	no
iHop	16204114	www.pdg.cnb.uam.es/UniPub/iHOP	yes	yes	single concept query	no[g]	yes[i]	no[g]	no	no	no	no	no	no
Retrieval of direct associations														
PubMatrix	14667255	pubmatrix.grc.nia.nih.gov/	no	yes	multiple PubMed queries	no	partly[b]	partly[b]	no	no	no	no	no	no
PubGene	11326270	www.pubgene.org/	yes	yes	multiple concepts	yes[h]	yes[h]	yes	no	no	no	no	no	no
Copub mapper	15760478	copub.gatcplatform.nl/	yes	yes	multiple gene concepts or a single other concept	no	no	yes[h]	no	no	no	no	yes[m]	no
Retrieval of direct and indirect associations														
Anni 2.0	-	www.biosemantics.org	yes	yes	multiple concepts	yes	yes	yes	yes	yes	yes	yes	yes	yes
TXTgate	15186494	tomcat.esat.kuleuven.be/txtgate/	yes	no[c]	multiple gene concepts	yes[h]	yes[h]	yes[h]	yes[h]	no	no	yes[h]	-	-
Knowledge discovery														
BITOLA	15694635	www.mf.uni-lj.si/bitola/	yes	yes	single concept query	no	no	no	no	yes	yes	no	no	yes[n]
Arrowsmith	16817965	arrowsmith.psych.uic.edu/arrowsmith_uic/	output[a]	yes	two document sets[d]	no	no	no	no	yes	yes	no	no	no
Biotermnet	2007, JASIST 58:51-65	btn.ontology.ims.u-tokyo.ac.jp/	yes	no[l]	multiple concepts	no	no	yes	yes	yes	yes	no	no	yes[n]
Manjal	2004, JASIST 55:396-413	sulu.info-science.uiowa.edu/Manjal.html	yes	no[c]	multiple concepts	yes[e]	no	no[k]	no[k]	yes	yes	no	-	-
